# Supplementary material for: Patterns of Intron Gain and Loss in Fungi
Source: PLoS Biol. 2004 Nov 30;2(12):e422. doi: 10.1371/journal.pbio.0020422 (PMC532390; doi:10.1371/journal.pbio.0020422)
Supplement: Table S1 — Also available at http://genes.mit.edu/NielsenEtAl/. (4.3 MB ZIP). [file pbio.0020422.st001.zip › NielsenEtAl/html/826.html]

AN7640.1.NCU05143.1.MG00194.1.FG03916.1


```
 CLUSTAL W (1.82) Multiple Sequence Alignments - Introns Inserted


Sequence 1: MG00194.1	468 aa
Sequence 2: FG03916.1	590 aa
Sequence 3: NCU05143.1	470 aa
Sequence 4: AN7640.1	486 aa
Alignment Length: 608 aa
Number Identitical Residues: 205 aa
Alignment Score (without introns) 10943


MG00194.1 	------------------------------------------------------------
NCU05143.1	------------------------------------------------------------
FG03916.1 	MACQTQSSMREGDTNHPDILLPRVCRSTKKNWRKVSIDIGDRHPNLGNRDKVDSSTVLSA
AN7640.1  	------------------------------------------------------------
          	                                                            

MG00194.1 	---------------~-----------------------------------------~--
NCU05143.1	---------------~-----------------------------------------~--
FG03916.1 	YGLTGVYGDSGRKAS0GSPFSFRANSSCFPGLQLQLPFAPRNGQRLRHDQVTNMTAK0AL
AN7640.1  	---------------~-----------------------------------------~MV
          	                                                            

MG00194.1 	~------MAPRSTLHYAGLLALASGSLAMPY-VSEAQTTVV-----SEAAITPPVVPHTN
NCU05143.1	~------MAGFKLSAGVGLLGLAARSLAVPAPIYSEPTTTF-----SVPKVTGVVEPVTN
FG03916.1 	2SISLQAMSTKSVLRSVALLAIAGTSYAAPF-VSEAQTTVS-----SSGVVAP-IRPYSD
AN7640.1  	~KLWSIATSNYTNRLNECLLDLAASAAALPTEVVKIPSVETQPPRGSEPGAAPTLVNSAT
          	 .    : :  .      ** :*. : * *  : .  :.  ... .*    :  :   : 

MG00194.1 	--VTGHGPYTGP--SPTTTGAIS-TSILASAVPVLPPPEDAYDYPADGELHGDQPAPYTP
NCU05143.1	--VTSHGPYLSMEATPTTTGALS-TSVLAPSVPALPPGPDAYKYPSDGQLHAPQPAPYTP
FG03916.1 	--VTSHGPYTG---TPTTTGAVS-TTILASAIPESPPPPGSYDYPADGKLHAPQPAPYTP
AN7640.1  	SGTTTHGPYTG---TPTTTGALQAESTLAQSIAPQPNPTATYYNP-DGRLTQPQPMPYMP
          	:..* **** .   :******:.: : ** ::.  *    :*  * **.*   ** ** *

MG00194.1 	SGGIWTNGSAPVYRVMSDFDYQSLALALYQEWIELDLFHWGLATYSDEEFEELGLNGEAR
NCU05143.1	AGGVGTNGSAPVYRVLSDFDYQSIALALHQEWIELDLFHWGLATFSVEDFEEAGLTAEDR
FG03916.1 	DGGVGTNGSAPVYRVQSDFDYQSLALALYQEWIELDLFRWGLATFSKEEWEAYGIGAEDR
AN7640.1  	AGGVGMNGTLPVYMVQSDFDYQSVALGVHQEYIELDLFHYGLERFSEQDFLDAGLTVEDR
          	 **:  **: *** * *******:**.::**:******::**  :* :::   *:  * *

MG00194.1 	HLIQHMAEQEVGHATVLTNMLGAQAPRQCTYNYPVSNLREYIDFNQKLTRWGEAGVYGFL
NCU05143.1	HLIEFMADQEVGHATLLSNILGPSAPNQCTYNYPATNVHEFIDFCQKLTRYGESGVYGFL
FG03916.1 	FLIEYMAEQEIGHATVISNMLGPQAPQQCTYNYPVSNVPEFIDFNQKLTRWGESGVYGFL
AN7640.1  	RLIEYMAIQEAGHATLLSNMLGESAPKQCTYNYPFTTVREFLDFNVKLTRWGESGNWGFL
          	 **:.** ** ****:::*:** .**.******* :.: *::**  ****:**:* :***

MG00194.1 	PHLNSGPAAQLLLQSITVEARQQMVFRQFEGLFPMPEWHTVG~IPQSWAWTLLAPYIASC
NCU05143.1	NHLNAHDVGQLLLQSITTEARQQMIFRQFEGLFPMPVWFEVG~IPQSWAWSLLAPYISSC
FG03916.1 	PHLNSGPAAQLLLQSITIEARQQLIFRQFGGNFPMPEWHTPG~IPQSWAWTLLAPYISSC
AN7640.1  	SHLDSKEVATLIVQAEAIEARQQSVFRQLLGLHPMPIWFAPG1VPQSWHWTLLAQYISSC
          	 **::  .. *::*: : ***** :***: * .*** *.  * :**** *:*** **:**

MG00194.1 	PWNQTRLIWQNFPALHVLNQPNPWRID-GSNVWNETT-GGWAN---TAETANITEEASCV
NCU05143.1	PENNTRLIWQNYPALNILNQPNPFRIN-GSYVWNETTDGGYAN---TASYQDIDESDDCT
FG03916.1 	PADQTRLVWQNFPSLNILNQPNPARID-GSNVWNETT-GGYAN---TLSTADVKKDELCV
AN7640.1  	PENNTRLVWQNFPNLYVTNQANPNRINPNTTEFNEISDASHVNRTGNPSSSHIPANESCV
          	* ::***:***:* * : **.** **:..:  :** :... .* :.. .  .:  .  *.

MG00194.1 	NATDPLQDCNAAISQNRSIPLSYAGRQVFLQWDAPGQPVGPNNSYITATEIHEPKFAAWV
NCU05143.1	KSTDVGVSCAPGITRNRSMPLSYPGRQVFLEWDEPGKPIGPNNSYVTDTQAGAPKFAAWV
FG03916.1 	NATDKTLDCPAAITNNRSIPLSYPGRQVFLNWDAPGQAVGPNNSYITSTNVKAPKFAAWV
AN7640.1  	HVNETGYNCGPAISRDKYEPLTFPGRKVFLTWDDPGRPVGPNNSYITSTTAGEPKFVAWV
          	: .:   .* ..*:.::  **::.**:*** ** **:.:******:* *    ***.***

MG00194.1 	SQLNVTYSPLLNVSLEDRTAYTIQPNVSTWEGDPAVNSTMFIALTDADVYVTPANLTMLN
NCU05143.1	SQLNVTYSPLEN--IQGNSAYTVQPDVSTFDGNPAINGTIFLVLTDEDLYVTPYNLTQLN
FG03916.1 	SQLNVTYSPLQNVSLEDRTAYTIQPNVSTWMGDPAINSTMFLALTDTDLYVTPYNLTMLN
AN7640.1  	SQLNTTYTELTT--TGKNEGFTFQPASEVYETDPALNGTAFIALTDSNPYVTPYNLTMLN
          	****.**: * .     . .:*.**  ..:  :**:*.* *:.*** : **** *** **

MG00194.1 	PHVAALAVYQAG
NCU05143.1	PHVYAVGMYQSG
FG03916.1 	PHVAALAVYQAG
AN7640.1  	PNVRALGLYQAG
          	*:* *:.:**:*
```
